# Prevalence and effect on prognosis of sarcopenia in patients with primary biliary cholangitis

**DOI:** 10.3389/fmed.2024.1346165

**Published:** 2024-02-29

**Authors:** Jiaqi Yang, Shuangshuang Jiang, Qingling Fan, Didi Wen, Yansheng Liu, Kemei Wang, Hui Yang, Changcun Guo, Xinmin Zhou, Guanya Guo, Yulong Shang, Ying Han

**Affiliations:** ^1^Department of Digestive Diseases, Xijing Hospital, Fourth Military Medical University, Xi’an, China; ^2^Department of Radiology, Xijing Hospital, Fourth Military Medical University, Xi’an, China

**Keywords:** sarcopenia, primary biliary cholangitis, prevalence, prognosis, nutrition

## Abstract

**Background:**

Sarcopenia adversely affects the treatment outcomes in Cirrhosis and NAFLD. However, such research is limited in primary biliary cholangitis (PBC) patients. This study was performed to examine the prevalence of sarcopenia and its impact on PBC patients’ prognoses.

**Methods:**

This study enrolled confirmed PBC patients who had an abdominal CT scan. Sarcopenia was determined by the L3-skeletal muscle index with a Chinese population-based cut-off value. Laboratory test values and liver stiffness measurements values were obtained from the electronic medical records.

**Results:**

In total, 174 PBC patients with a median age of 54 (IQR, 48, 62) years old, were enrolled. 45 (25.9%) patients among them were diagnosed with sarcopenia. Univariate and multivariate logistic regression results illustrated that male gender (OR = 9.152, 95%CI = 3.131–26.751, *p* < 0.001) and LSM ≥ 12.8 kPa (OR = 4.539, 95%CI = 1.651, 12.478, *p* = 0.003) were the independent risk factors of sarcopenia in PBC patients. In the prognosis analysis, sarcopenia was determined as a risk factor for indicating adverse events in PBC patients (HR = 4.058, 95%CI = 1.955–8.424, *p* < 0.001) by Cox proportional hazards regression.

**Conclusion:**

The current findings illustrate that comprehensive evaluation and management of sarcopenia may contribute to the improvement of treatment outcomes and life quality of PBC patients.

## 1 Introduction

Primary biliary cholangitis (PBC) is an autoimmune induced chronic cholestatic liver disease characterized by non-suppurative inflammation of the small bile ducts inside the liver (1, 2). Frailty and pruritus as the two most frequent complaints from PBC patients occur in 50 to 78% of the patients with PBC (3, 4). The only first-line drug for PBC is ursodeoxycholic acid (UDCA) and it could effectively improve patients’ survival (5). The second-line drugs such as obeticholic acid and fibrates can be used when patients inadequately respond to UDCA (6, 7). However, there is still a subgroup of patients with PBC who face a high risk of rapidly progressing into cirrhosis and liver failure. To better manage these patients, simple and reliable non-invasive methods, for example, the GLOBE score and UK score have therefore been developed to monitor PBC patients’ progression (8–10).

Recently, sarcopenia has been detected as a prognostic indicator in a series of diseases such as cancers, heart failure and NAFLD (11, 12). Sarcopenia, defined as a decline in skeletal muscular strength and mass, is a condition that is highly prevalent in a variety of chronic diseases (13). Sarcopenia is present in estimated 40–70% of cirrhosis patients, and cirrhosis patients accompanied with sarcopenia face higher risks of mortality, decompensation, and a lower life quality (14).

As for PBC, only one research showed that approximately 23.1% of PBC patients have sarcopenia (15). However, these studies did not have follow-up data, and the impact of sarcopenia on the prognosis of PBC patients is still unclear. Therefore, this retrospective study was performed to determine sarcopenia prevalence and its related characteristics among patients with PBC. Additionally, this study aimed to assess the influence of sarcopenia on the prognosis of patients with PBC.

## 2 Patients and methods

### 2.1 Patients

PBC patients aged ≥18 years who were hospitalized in Xi Jing Hospital of the Fourth Military Medical University (Xi’an, China) and had an abdominal CT scan between August 2015 to December 2022 were retrospectively recruited into the current study. To be diagnosed with PBC, patients must satisfy a minimum of two of the three criteria listed below: (i) Biochemical evidence of cholestasis, (ii) AMA positive or sp100, gp210 positive, (iii) Histological features of PBC. All the patients were regularly treated with UDCA or UDCA combined with Fenofibrate. Patients were excluded for the following criteria: (1) Presence of other liver diseases, such as alcoholic liver disease, primary sclerosing cholangitis, and viral hepatitis (hepatitis B or C); (2) Patients with malignancy; (3) Patients who underwent liver transplantation or suffered from the complications of cirrhosis (hepatic encephalopathy, variceal bleeding, or ascites) before the date of baseline.

### 2.2 Clinical data

The baseline of this study was defined as the date of the CT scans. The baseline Clinical characteristics and laboratory test values were collected from the electronic medical records. As for those patients for whom the data were not available on the date of CT scans, laboratory data close to the date of the CT scans but no more than 2 months were obtained. We also collected the LSM data assessed by vibration-controlled transient elastography (VCTE) within 6 months of the date of CT scans. The diagnosis of cirrhosis is according to the CT images and laboratory test results. The primary outcomes were the occurrence of adverse events, including liver transplantation, liver-related death and cirrhosis-related complications (variceal bleeding, hepatic encephalopathy, or ascites). Survival was determined from the date of CT scans to the date of occurring primary outcomes to evaluate the impact of sarcopenia on PBC patients. The data were censored at the time last follow-up for the living patients.

### 2.3 Assessment of sarcopenia in patients with PBC

Slice-O-Matic software (5.0, Tomovision, Milletta, QC, Canada) was used to analyze lumbar level 3 (L3) slice CT scans. Tissue boundaries were edited by one trained researcher who was blinded to patient outcomes. Total skeletal muscle area included the psoas, rectus abdominus, internal/external oblique, quadratus lumborum, and erector (as shown in Supplementary Figure S1). Total skeletal muscle area divided by the square of height then yielding the skeletal muscle index (SMI) in cm^2^/m^2^. According to a Chinese population-based multicenter study, the L3-SMI cutoff values for sarcopenia were set as 44.7 cm^2^/m^2^ and 32.5 cm^2^/m^2^ in men and women, respectively (16), to define sarcopenia.

### 2.4 Statistical analysis

Continuous variable data were shown as median with interquartile range (IQR), and comparisons of these data were performed using the Wilcoxon rank test, while counts with percentages were presented for categorical variables, and Chi-squared tests or Fisher’s exact tests were implemented for comparisons. Sarcopenia-associated factors among PBC patients were identified by logistic regression analysis. Adverse events-free survival was estimated via the Kaplan–Meier method, and a log-rank test was adopted for comparison. Other variates were adjusted using the Cox proportional hazards regression model. SPSS software (version 26.0; SPSS Inc., Chicago, IL, USA) and R software (version 4.0) were utilized to analyze all statistical data. The statistical significance level was established at a two-sided *p* value of <0.05.

## 3 Results

### 3.1 Characteristics of the patients

**Figure 1:**
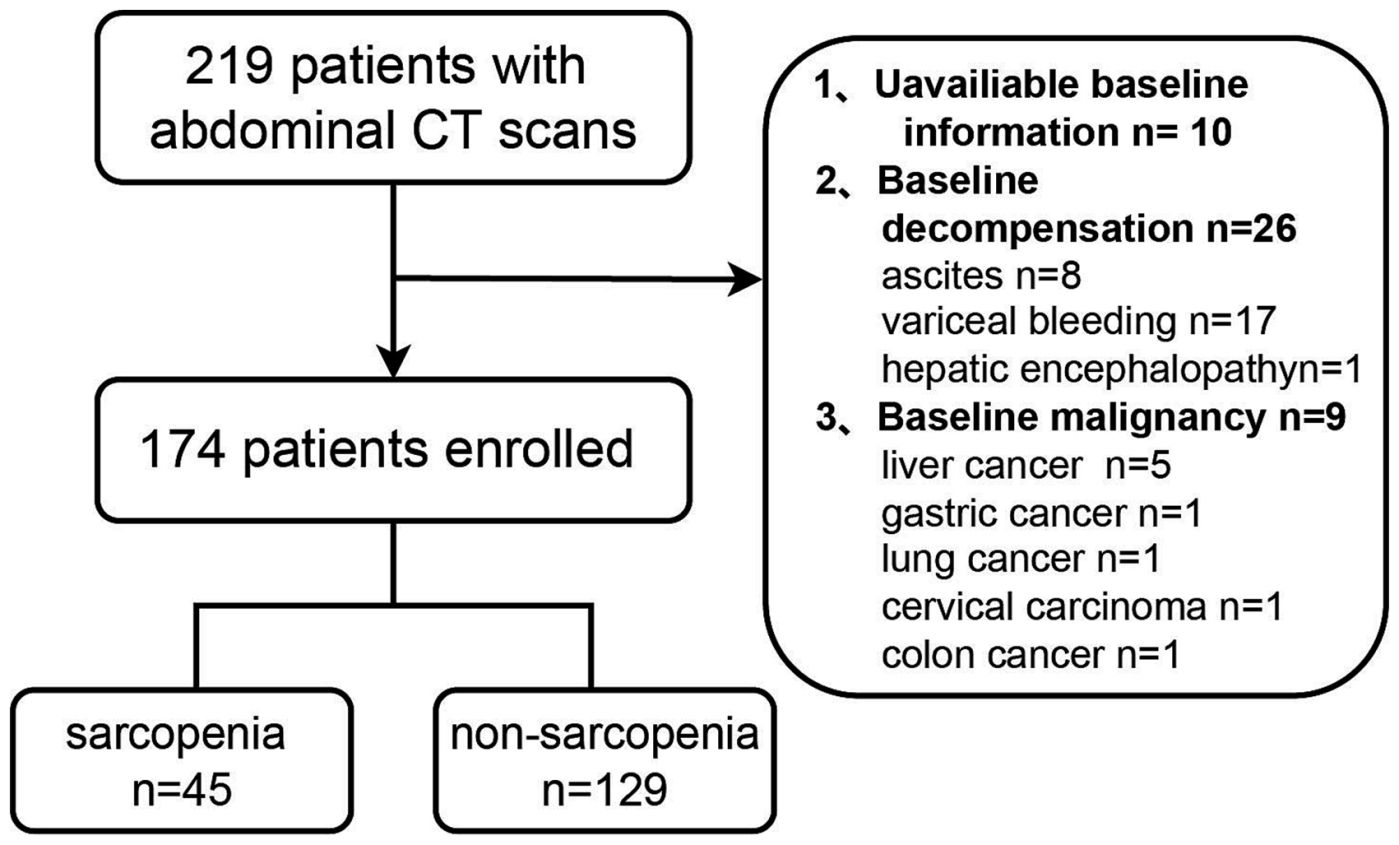
Flow chart of the study.

The study included 174 participants with PBC, with a median age of 54 (IQR, 48, 62) years. Among them, 147 (84.5%) patients were female, and 150 (86.2%) patients were AMA-positive. The flow chart is shown in Figure 1. The main reason for patients submitted to CT is for evaluation of liver cirrhosis and portal vein condition (106, 60.8%), other reasons are shown in Supplementary Table S1.

### 3.2 Baseline characteristics of sarcopenia vs. non-sarcopenia in patients with PBC

Among the 174 patients, 45 (25.9%) were diagnosed with sarcopenia according to the definition of sarcopenia by L3-SMI. Demographic and laboratory data were compared between those with sarcopenia and non-sarcopenia patients (Table 1). The median age of the sarcopenia group was 57 years (IQR, 52, 63), whereas that of the non-sarcopenia group was 54 years (IQR, 48, 62, *p* = 0.016). Significant variations were observed in the SMI between the two groups, the median SMI of the sarcopenia group was 31.17 cm^2^/m^2^ (IQR, 29.53, 33.37), while that of the non-sarcopenia group was 38.84 cm^2^/m^2^ (IQR, 36.24, 42.79, *p* < 0.001). Significant variations were also found between the sarcopenia group and the non-sarcopenia group in RBC levels (3.76 vs. 4.05, *p* = 0.013), but there was no significant variation in terms of HGB (109 vs. 120, *p* = 0.101). Additionally, Sarcopenia and non-sarcopenia groups differed significantly in terms of TBIL and LSM levels (all *p* < 0.05). Interestingly, the rate of sp100 positive in the sarcopenia group (22.9%) was higher than that in the non-sarcopenia group (7%), with a significant difference (*p* = 0.015).

**Table 1:** Baseline clinical characteristics of patients with and without sarcopenia.

Number (%)/Median (IQR)	Overall *n* = 174	Non-sarcopenia *n* = 129	Sarcopenia *n* = 45	*P*
Time of using UDCA, month	18.50 [0.00, 49.75]	17.00 [0.00, 51.00]	21.00 [0.00, 36.00]	0.38
Female (%)	147 (84.5)	117 (90.7)	30 (66.7)	<0.001
Age, year	54.00 [48.00, 62.75]	54.00 [48.00, 62.00]	57.00 [52.00, 63.00]	0.128
SMI, cm^2^/m^2^	37.78 [34.01, 41.99]	38.84 [36.24, 42.79]	31.17 [29.53, 33.37]	<0.001
WBC, 10^9^/L	4.14 [3.37, 5.66]	4.20 [3.36, 5.80]	3.92 [3.41, 4.84]	0.387
RBC, 10^12^/L	3.94 [3.51, 4.35]	4.05 [3.64, 4.39]	3.76 [3.38, 4.25]	0.013
HGB, g/L	120.00 [100.0, 133.75]	120.00 [104.0, 135.0]	109.00 [91.0, 132.0]	0.101
PLT, 10^9^/L	124.00 [76.5, 189.50]	130.00 [80.00, 194.00]	107.00 [66.0, 161.0]	0.117
ALT, ULN	1.23 [0.56, 10.25]	1.32 [0.59, 12.00]	1.04 [0.50, 2.30]	0.221
AST, ULN	1.74 [0.88, 18.00]	1.85 [0.94, 22.00]	1.43 [0.88, 3.33]	0.402
ALB, g/L	38.80 [35.23, 42.48]	39.80 [35.70, 42.70]	37.20 [34.30, 40.90]	0.035
TBIL, ULN	0.87 [0.60, 1.41]	0.76 [0.57, 1.24]	1.06 [0.78, 2.08]	0.007
ALP, ULN	1.31 [0.86, 2.10]	1.23 [0.82, 2.02]	1.43 [0.94, 2.48]	0.278
GGT, ULN	5.02 [1.53, 18.0]	5.30 [1.62, 18.56]	4.25 [1.50, 9.38]	0.409
VD, nmol/L	29.61 [19.23, 47.75]	30.67 [20.86, 45.60]	23.98 [15.02, 63.28]	0.579
INR	1.00 [0.94, 1.09]	0.98 [0.94, 1.08]	1.05 [0.96, 1.10]	0.111
IgG, g/L	15.00 [11.90, 18.0]	14.20 [11.70, 17.70]	16.15 [13.35, 18.60]	0.164
IgM, g/L	2.63 [1.75, 4.07]	2.48 [1.75, 3.68]	2.99 [1.84, 4.81]	0.233
LSM, kPa	12.25 [9.65, 17.09]	11.61 [7.99, 15.08]	16.77 [12.02, 19.82]	0.001
Cirrhosis	95 (54.6)	60 (46.5)	35 (77.8)	0.001
AMA (%)	150/174 (86.2)	108/129 (83.7)	42/45 (93.3)	0.993
AMA-M2 (%)	113 /158 (71.5)	85/126 (67.5)	28/32 (87.5)	0.043
gp210 (%)	54/164 (32.9)	43/129 (33.3)	11/35 (31.4)	0.992
sp100 (%)	17/164 (10.4)	9/129 (7.0)	8/35 (22.9)	0.015

ALP, alkaline phosphatase; ALB, albumin; AST, aspartate aminotransferase; ALT, alanine transaminase; GGT, gamma-glutamyl transferase; TBIL, total bilirubin; INR, international normalized ratio; IQR, interquartile range; PLT, platelet; RBC, red blood cell; IgM, immunoglobulin M; IgG, immuno-globulin G; HGB, hemoglobin; AMA, anti-mitochondrial antibody; AMA-M2, M2 subtype of an-ti-mitochondrial antibody.

### 3.3 The correlation of SMI with other variates

**Figure 2:**
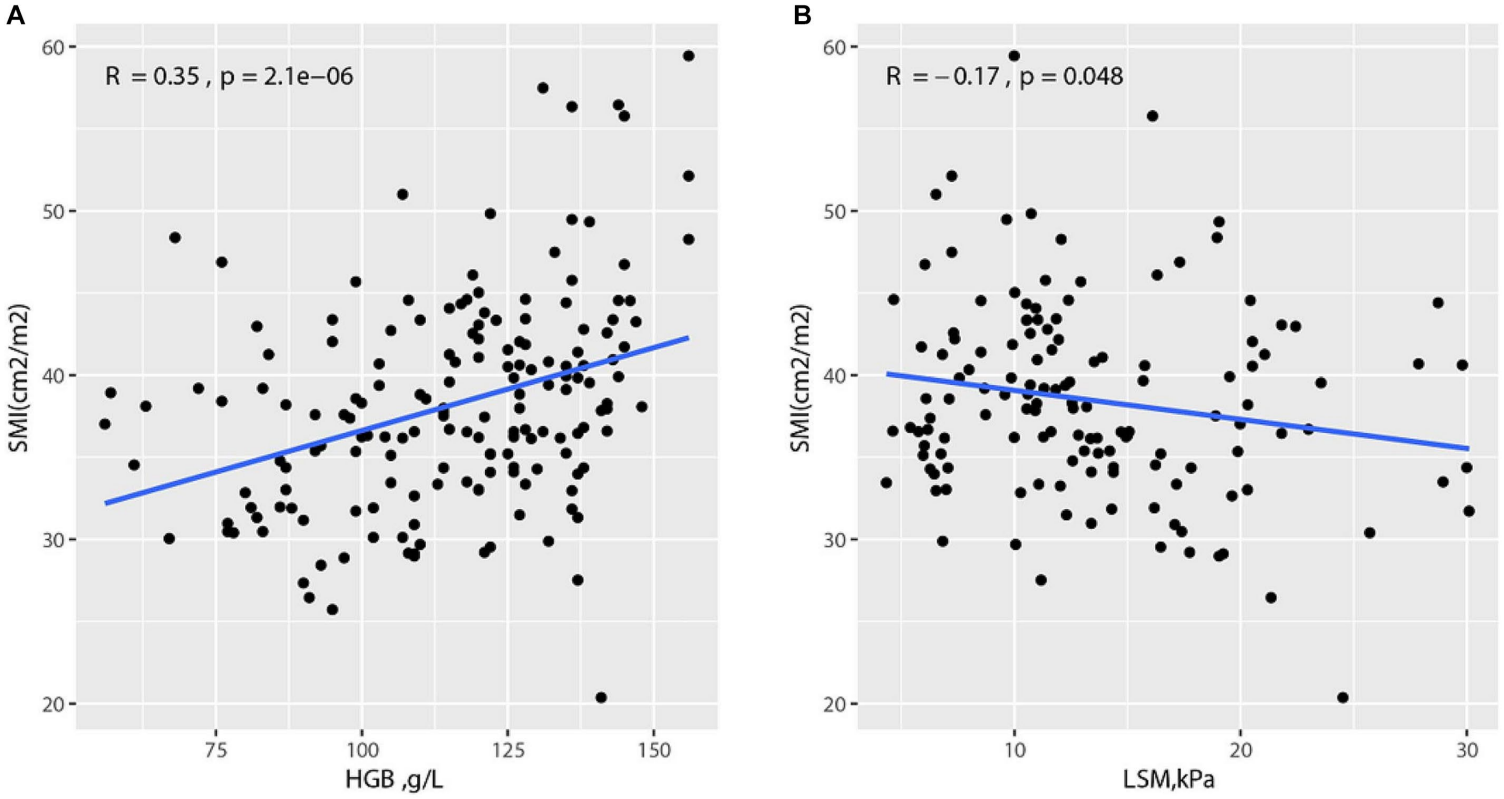
Correlation between SMI and other variates **(A)** Correlation between SMI and HGB; **(B)** Correlation between SMI and LSM.

To explore the correlation between SMI and other variates, Spearman’s rank correlation test was conducted. The results are shown in Supplementary Figure S2, and SMI was negatively correlated with LSM (*R* = −0.17, *p* = 0.048). Additionally, SMI and HGB were positively correlated (*R* = 0.35, *p* < 0.001, Figure 2).

### 3.4 Factors associated with sarcopenia in patients with PBC

The factors associated with sarcopenia in PBC patients were assessed using univariate and multivariate logistic regression (Table 2). The result of univariate logistic regression showed that male gender (OR = 4.875, 95%CI = 2.066–11.504, *p* < 0.001), anemia (male HGB ≤ 120 g/L, female HGB ≤ 110 g/L) (OR = 2.964, 95%CI = 1.468–5.984, *p* = 0.02), TBIL≥1ULN (OR = 3.278, 95%CI = 1.141–4.547, *p* = 0.02) and LSM ≥12.8 kPa (OR = 3.988, 95%CI = 1.612–9.869, *p* = 0.003) were the risk factors of sarcopenia in PBC patients. These variates as well as age were further subjected to the multivariate logistic regression model. Here, we found that male gender (OR = 9.152, 95%CI = 3.131–26.751, *p* < 0.001) and LSM ≥ 12.8 kPa (OR = 4.539, 95%CI = 1.651, 12.478, *p* = 0.003) were the independent risk factors of sarcopenia in PBC patients.

**Table 2:** Factors associated with sarcopenia in patients with primary biliary cholangitis.

Variable	Univariate		Multivariate	
	*P*	OR (95% CI)	*P*	OR (95% CI)
Age ≥65y vs. < 65y	0.772	1.112 (0.54–2.293)		
Gender Male vs. female	<0.001	4.875 (2.066–11.504)	<0.001	9.152 (3.131–26.751)
Anemia vs. non-Anemia^a^	0.02	2.964 (1.468–5.984)		
PLT ≤100×109/L vs. >100×109/L	0.478	1.283 (0.644–2.558)		
AST ≥2ULN vs. <2ULN	0.177	0.616 (0.305–1.244)		
TBIL ≥1ULN vs. <1ULN	0.02	2.278 (1.141–4.547)		
ALP ≥1.67ULN vs. <1.67ULN	0.413	1.333 (0.67–2.654)		
LSM ≥12.8 kPa vs. <12.8 kPa	0.003	3.988 (1.612–9.869)	0.003	4.539 (1.651, 12.478)

^a^Anemia was defined by male HGB ≤120 g/L, female HGB ≤110 g/L.

### 3.5 Impact of sarcopenia on the adverse events in patients with PBC

We collected follow-up information of 174 patients, and the median follow-up time is 24 months (IQR, 14, 39). Specifically, 40 patients suffered from adverse events, 5 patients suffered liver-related death or liver transplantation, 18 patients developed ascites, 14 patients had variceal bleeding and 3 patients developed hepatic encephalopathy.

**Figure 3:**
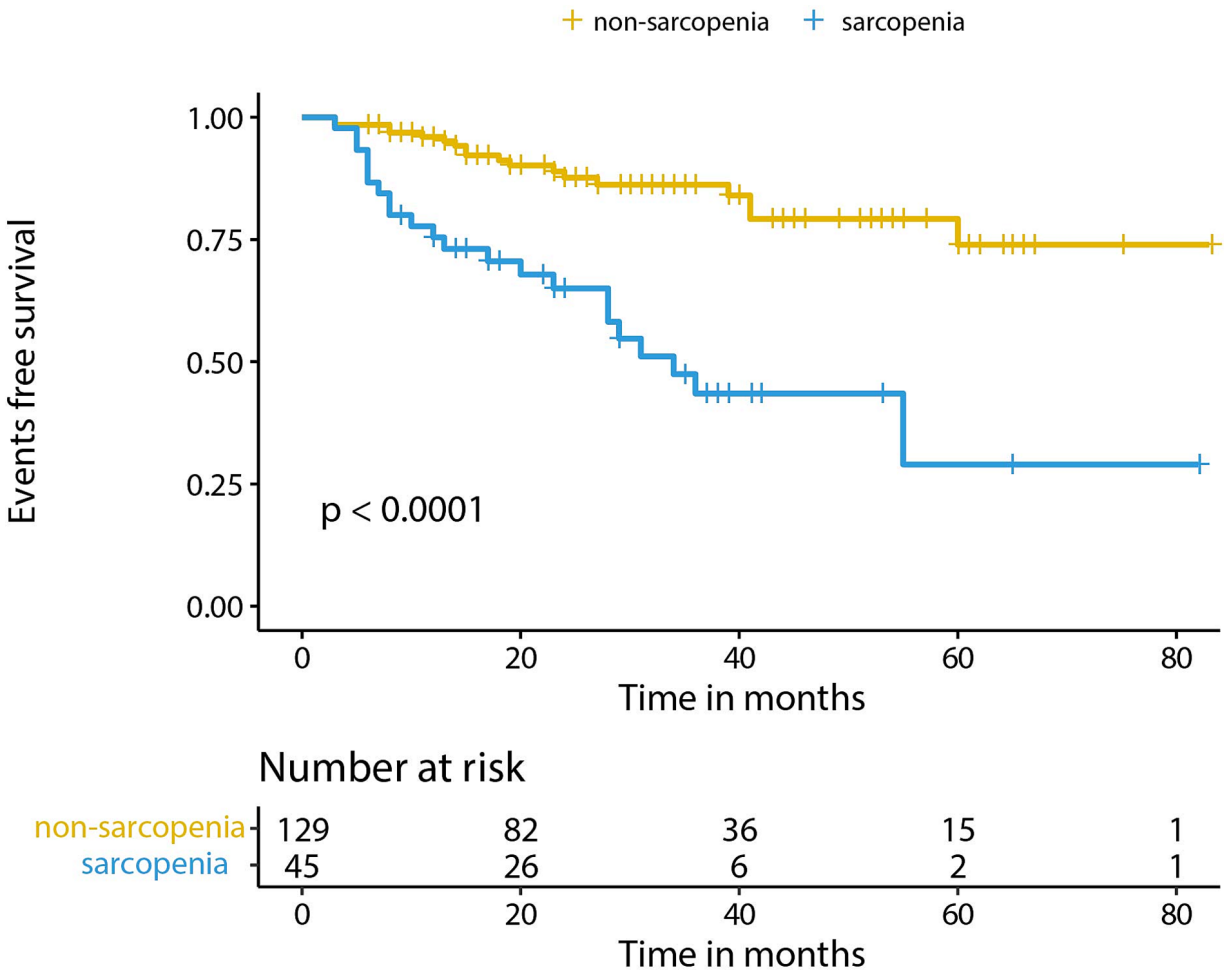
The events free survival of PBC patients with sarcopenia vs. non-sarcopenia.

According to the Kaplan–Meier survival analysis (Figure 3), the events-free survival rate of sarcopenic patients was remarkably lower in comparison with that of non-sarcopenic patients (*p* < 0.001). We performed subgroup analysis by dividing PBC patients into two groups: the cirrhosis group and the non-cirrhosis group. Compared to patients without sarcopenia, those with liver cirrhosis had a remarkably lower event-free survival rate (*p* < 0.001), while there was no significant difference between the two groups of patients in the non-cirrhosis group (Supplementary Figure S3).

To determine whether sarcopenia was an independent factor correlated with the adverse events, we first performed a univariate Cox proportional hazards regression model without adjusting any covariant, and the result confirmed sarcopenia as a risk factor for adverse events in PBC patients (HR = 4.198, 95% CI = 2.243–7.857, *p* < 0.001). Then we adjusted age and gender, and the result was also significant (HR = 4.812, 95%CI = 2.501–9.260, *p* < 0.001). Finally, a multivariate Cox proportional hazards regression model was conducted in steps adjusting several covariates, namely cirrhosis, ALP, PLT, INR, TBIL, and ALB (Table 3). These were confirmed to influence the outcomes of PBC patients. After that, the result was still solid, which indicated that sarcopenia independently functioned as a risk factor for adverse events in PBC patients (HR = 4.058, 95%CI = 1.955–8.424, *p* < 0.001). We also conducted subgroup analysis in patients with cirrhosis and the results (Supplementary Table S2) showed that Sarcopenia is an independent risk factor for adverse events in PBC patients with cirrhosis (HR = 2.290, 95% CI = 1.024–5.121, *p* = 0.043).

**Table 3:** Hazard ratio for adverse events in patients with sarcopenia vs. non-sarcopenia.

	β	*P*	HR	95%CI
Unadjusted	1.435	<0.001	4.198	[2.243–7.857]
Adjusted by Age and gender	1.571	<0.001	4.812	[2.501, 9.260]
Plus Cirrhosis	1.408	<0.001	4.087	[2.107, 7.929]
Plus ALP	1.410	<0.001	4.096	[2.11, 7.951]
Plus PLT	1.403	<0.001	4.069	[2.070, 8.00]
Plus INR	1.421	<0.001	4.140	[2.038, 8.412]
Plus TBIL	1.422	<0.001	4.147	[1.990, 8.643]
Plus ALB^*^	1.401	<0.001	4.058	[1.955, 8.424]

^*^The final Cox proportional hazard model included Sarcopenia, Age, Gender, Cirrhosis, ALP, PLT, INR, TBIL, and ALB.

## 4 Discussion

Extensive research has indicated that sarcopenia is linked to increased decompensation and even death in patients with cirrhosis and NAFLD (17–19), but these studies are limited to PBC. Therefore, the current retrospective study was performed to address such a gap. The analysis indicated that sarcopenia was prevalent among PBC patients at a prevalence rate of 25.9%. Additionally, the univariate and multivariate logistic regression indicated male and LSM ≥ 12.8 kPa as independent factors associated with sarcopenia in patients with PBC. Furthermore, this study revealed that PBC patients combined with sarcopenia showed an increased risk of adverse events. These findings presented here could improve our understanding of the association between PBC and sarcopenia.

It has been reported that 40 to 70% of patients with cirrhosis and end-stage liver diseases combined with sarcopenia (14). Most of the PBC patients in our sample did not progress to cirrhosis, with cirrhosis patients constituting 54.6%. Therefore, a higher proportion of PBC patients may be combined with sarcopenia in the real world.

Although the mechanism of PBC patients combining sarcopenia is still elusive, there are several potential mediators of the liver-muscle axis contributing to sarcopenia including hyperammonemia and endotoxemia. Ammonia plays a vital role in the liver-muscle axle, however, the ammonia disposal process could be impaired due to hepatocyte dysfunction and up-taking of ammonia in skeletal muscle as a result of portosystemic shunting (20, 21). In addition, a recent analysis indicated that hyper-ammonemia could impair mTORC1 signaling and increase phosphorylation of eukaryotic initiation factor 2α, resulting in decreased muscle protein synthesis (22). Systemic endotoxemia activates Toll-like receptors expressed on muscle and increases proteolysis, both of which contribute to sarcopenia as a result of the alterations in the gut microbiome and disruption of the gastrointestinal mucosal barrier (23). Additionally, increased circulation of IL-6, TNF-α, and other inflammation factors in patients with PBC are also involved in dysregulated protein homeostasis and sarcopenia (24).

As an autoimmune disease, PBC also has some special relationship with sarcopenia. Firstly, a high prevalence of osteoporosis in patients with PBC has been reported and many researchers reporting osteoporosis as an independent risk factor for sarcopenia (15, 25). Furthermore, in 80% of patients with PBC, serum lipids are elevated. Although there is no increased mortality risk associated with atherosclerosis and cardiovascular diseases for patients with PBC, the high lipid levels could have adverse effects on muscle mass and function (26).

In our study, LSM ≥12.8 kPa was a risk factor for sarcopenia in patients with PBC in univariate logistic regression and multivariate analysis. Various studies have demonstrated that LSM is the most effective surrogate marker for predicting unfavorable outcomes in PBC patients and for detecting cirrhosis or severe fibrosis. Recent research has linked sarcopenia to severe liver fibrosis in NAFLD patients (27). These results indicated that liver fibrosis may have a close relationship with sarcopenia.

Moreover, we discovered that the HGB level was strongly linked to SMI, and anemia (male HGB ≤ 120 g/L, female HGB ≤ 110 g/L) is a risk factor for sarcopenia in patients with PBC in univariate logistic regression but not in multivariate analysis. However, Akihiko et al. showed that HGB < 109 g/L in females and HGB < 124 g/L in males independently functioned as a risk factor for sarcopenia in cirrhosis patients (28). In addition, previous studies have reported that HGB level is correlated with skeletal muscle mass in older adults (29) and low HGB is involved in the development of sarcopenia in those aged ≥60 years (30). Though the underlying mechanism is unclear, these findings indicate a strong link between sarcopenia and HGB in PBC patients. Future research is needed to fully illustrate the relation.

ALP and TBIL have always been the most widely used predictors of PBC patient outcomes (2). A recent study demonstrated that TBIL levels ≤0.6 ULN and normalized ALP are correlated with the lowest risk of dying or requiring liver transplants in PBC patients (31). Our study revealed a novel predictor of outcomes of patients with PBC. The current results demonstrated that the risk of suffering from adverse events in sarcopenia patients is much higher than in non-sarcopenia patients, indicating that sarcopenia could act as a complementation to the laboratory variate predictors.

Over 50% of patients with PBC experience frailty and pruritus, and their symptom burden is broad and significant. Frailty includes both mental frailty and physical frailty (32). Mental frailty refers to an individual who is frail and is vulnerable to poor recovery from a stressor event. As a subset of frailty, physical frailty is characterized by exhaustion, unintentional weight loss, sluggish gait, weakness, and inadequate physical exercise (33, 34). Therefore, Sarcopenia and physical frailty are closely related, and sarcopenia could account for some of the physical frailty of patients with PBC.

The prognosis of PBC patients and response to UDCA may be affected by the symptoms themselves, as a previous study (3) found that there is an association between severe pruritus and non-responsiveness to UDCA. Slimily one research study also indicated that the frailty phenotype appears to be stable in PBC, and the presence of frailty increases mortality risk significantly (35). These results indicated that the management of sarcopenia could improve frailty symptoms and therefore the treatment outcomes and quality of life for PBC patients.

Physical activity and nutrition interventions are the two dominant treatments for sarcopenia. Evidence for its benefits in sarcopenia is compelling. For instance, numerous studies have demonstrated the importance of adequate protein and nutrient intake and exercise in maintaining muscle mass and strength (36, 37). Specifically, vitamin D could promote the proliferation and differentiation of myogenic cells in the muscles and increase muscle mass directly. According to a recent study, vitamin D supplementation promotes strength and physical performance in patients with lower baseline vitamin D levels (38).

To our knowledge, this is the first research that shows the effect of sarcopenia on PBC patients’ outcomes. The effects of sarcopenia could include decreased functionality, which could lead to disability, loss of independence, and inability to perform daily activities (39). Our results may shed light on the comprehensive management of PBC patients not only in terms of drug use but also in terms of exercise and nutrition intake.

Despite the study’s strengths, there are still some limitations. Firstly, the decompensated cirrhosis patients were excluded, which may lead to some bias in patients’ selection. Secondly, the sample size of this study was relatively small. Lastly, owing to the limitations of the retrospective study, we were unable to clarify the relationship between anemia, sarcopenia, and PBC. Therefore, a large-scale, prospective, and multi-center study is needed to fully illustrate the relationship between the two.

## 5 Conclusion

In conclusion, sarcopenia has a relatively high prevalence among patients with PBC and has a close relationship with LSM and the male gender. Full assessment and treatment for sarcopenia may contribute to the improvement of treatment outcomes and life quality of PBC patients.

## Data availability statement

The original contributions presented in the study are included in the article/Supplementary material, further inquiries can be directed to the corresponding authors.

## Ethics statement

This study design was approved by the ethics committee of the Xijing Hospital of the Air Force Military Medical University (KY20151230-5). The studies were conducted in accordance with the local legislation and institutional requirements. Written informed consent for participation was not required from the participants or the participants’ legal guardians/next of kin in accordance with the national legislation and institutional requirements.

## Author contributions

JY: Data curation, Formal analysis, Writing – original draft. SJ: Methodology, Writing – original draft. QF: Methodology, Writing – original draft. DW: Data curation, Writing – original draft. YL: Methodology, Writing – original draft. KW: Methodology, Software, Writing – original draft. HY: Data curation, Methodology, Writing – original draft. CG: Supervision, Writing – review & editing. XZ: Writing – review & editing. GG: Writing – review & editing. YS: Supervision, Writing – review & editing. YH: Funding acquisition, Supervision, Writing – review & editing.
