# Supplementary material for: Prevalence and effect on prognosis of sarcopenia in patients with primary biliary cholangitis
Source: Front Med (Lausanne). 2024 Feb 29;11:1346165. doi: 10.3389/fmed.2024.1346165 (PMC10937409; doi:10.3389/fmed.2024.1346165)
Supplement: Supplementary file 1 [file Data_Sheet_1.docx]

Supplementary Material

Prevalence and effect on prognosis of sarcopenia in patients with primary biliary cholangitis

Jiaqi Yang,^1^* Shuangshuang Jiang,^1^* Qingling Fan,^1^* Didi Wen,^2^ Yansheng Liu,^1^ Kemei Wang,^1^ Hui Yang,^1^ Changcun Guo,^1^ Xinmin Zhou,^1^ Guanya Guo,^1^ Yulong Shang,^1^ Ying Han ^1^

Corresponding author:

Guanya guo: Xijing Hospital of Digestive Diseases The Fourth Military Medical University Xi’an, 710032, Shaanxi, China E-mail: guoguanya@126.com

Yulong Shang: Xijing Hospital of Digestive Diseases The Fourth Military Medical University Xi’an, 710032, Shaanxi, China E-mail: shangyul870222@163.com

Ying Han: Xijing Hospital of Digestive Diseases The Fourth Military Medical University Xi’an, 710032, Shaanxi, China E-mail: hanying1@fmmu.edu.cn


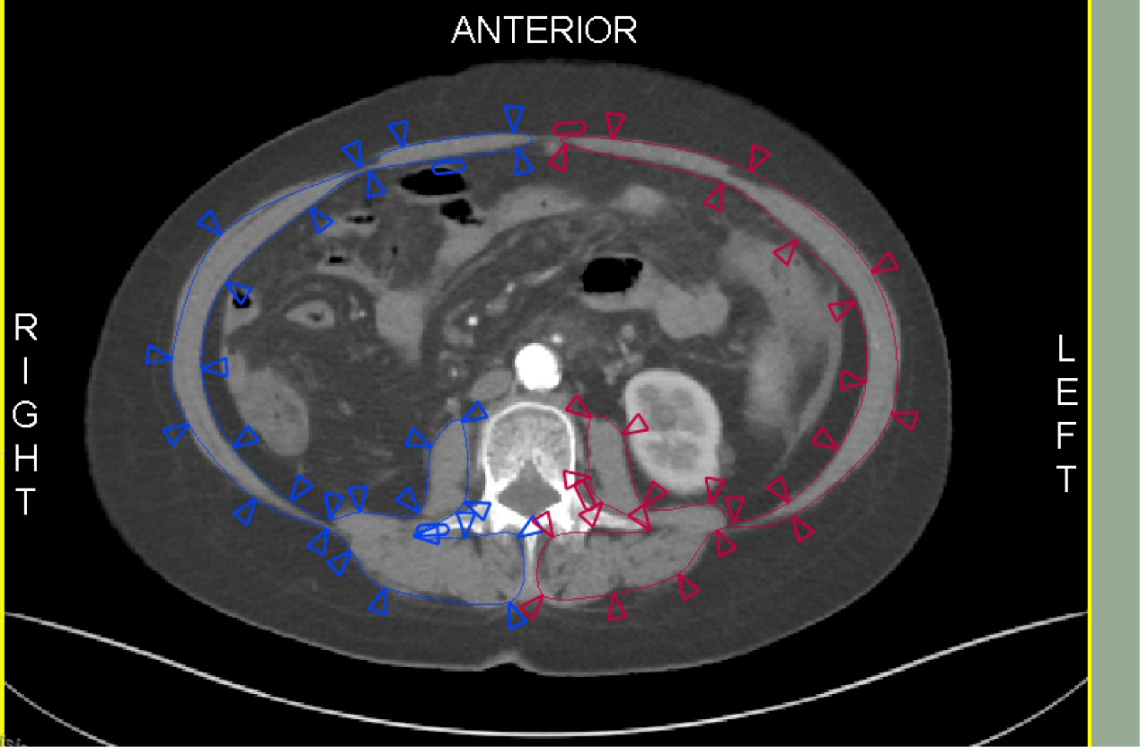


Supplementary Fig.1 Example of measurement of total muscle area at the level of L3 vertebra


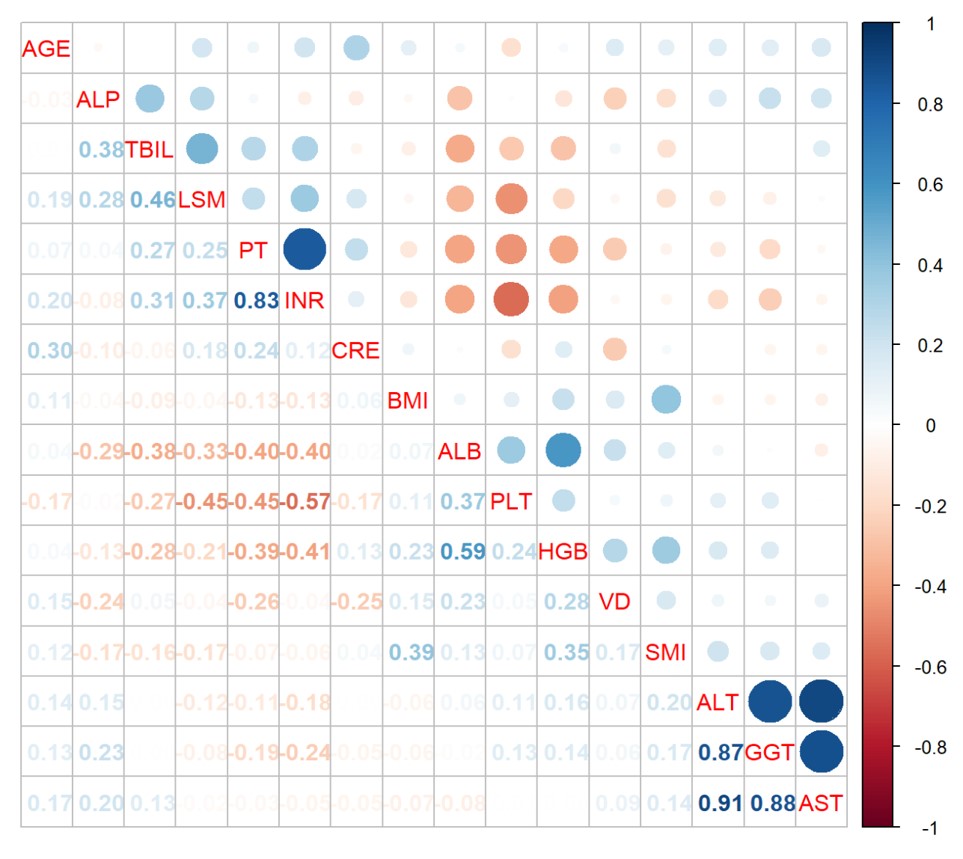


Supplementary Fig.2 The correlation between SMI and other variates

| 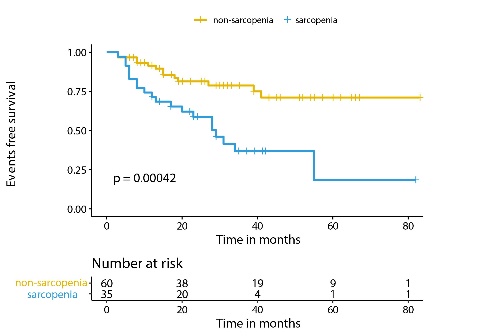 | 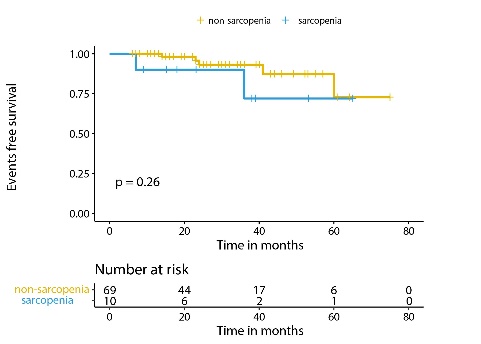 |
| --- | --- |
| (**a**) | (**b**) |

Supplementary Fig.3 The subgroup analysis of events free survival of PBC patients with sarcopenia vs. non-sarcopenia (**a**) patients with cirrhosis; (**b**) patients with non-cirrhosis

Supplementary Table1. Reasons of patients submitted to CT scan

| Number of patients (%) | Reason |
| --- | --- |
| 106 (60.8%) | Evaluation of liver cirrhosis and portal vein condition |
| 54 (31.3%) | Differential diagnosis, excluding other diagnoses |
| 10 (5.7%) | Hepatic Occupied Disease |
| 2 (1.1%) | Elevated alpha fetoprotein |
| 2 (1.1%) | Lumbar disc herniation |

Supplementary Table2. Hazard ratio for adverse events in cirrhosis patients with sarcopenia vs. non-sarcopenia

| Variable | Univariate | | Multivariate | |
| --- | --- | --- | --- | --- |
|  | *P* | HR (95% CI) | *P* | HR (95% CI) |
| Age  ≥65y vs.<65y | 0.814 | 1.088 (0.537 -2.204 ) |  |  |
| Gender  male vs. female | 0.904 | 1.059 (2.066- 2.750 ) |  |  |
| Sarcopenia vs. non-Sarcopenia | 0.020 | 2.356 (0.885- 4.863 ) | 0.043 | 2.290 (1.024- 5.121 ) |
| PLT  ≤100x109/L vs. >100x109/L | 0.003 | 3.141(1.142-6.748) | 0.023 | 2.736(1.146-6.533) |
| AST  ≥2ULN vs. <2ULN | 0.910 | 0.961(0.489-1.891) |  |  |
| TBIL  ≥1ULN vs. <1ULN | 0.02 | 2.278(1.141-4.547) | 0.006 | 3.055(1.368-6.823) |
| ALP  ≥1.67ULN vs. <1.67ULN | 0.805 | 0.916(0.457-1.834) |  |  |
| INR  ≥1.1vs. <1.1 | 0.013 | 2.450(1.208-4.971) |  |  |
